# Decreased radiation doses to tongue with “stick-out” tongue position over neutral tongue position in head and neck cancer patients who refused or could not tolerate an intraoral device (bite-block, tongue blade, or mouthpiece) due to trismus, gag reflex, or discomfort during intensity-modulated radiation therapy

**DOI:** 10.18632/oncotarget.10621

**Published:** 2016-07-16

**Authors:** Whoon Jong Kil, Christina Kulasekere, Ronald Derrwaldt, Jacob Bugno, Craig Hatch

**Affiliations:** ^1^ Cleveland Veterans Affairs Medical Center, Cleveland, Ohio, USA

**Keywords:** head and neck cancer, IMRT, tongue position, customized immobilization mask, oral cavity

## Abstract

**Purpose:**

To assess changes in oral cavity (OC) shapes and radiation doses to tongue with different tongue positions during intensity-modulated radiation therapy (IMRT) in patients with head and neck squamous cell carcinoma (HNSCC) but who refused or did not tolerate an intraoral device (IOD), such as bite block, tongue blade, or mouthpiece.

**Results:**

Tongue volume outside of OC was 7.1 ± 3.8 cm^3^ (5.4 ± 2.6% of entire OC and 7.8 ± 3.1% of oral tongue) in IMRT-S. D_mean_ of OC was 34.9 ± 8.0 Gy and 31.4 ± 8.7 Gy with IMRT-N and IMRT-S, respectively (*p* < 0.001). OC volume receiving ≥ 36 Gy (V36) was 40.6 ± 16.9% with IMRT-N and 33.0 ± 17.0% with IMRT-S (*p* < 0.001). D_mean_ of tongue was 38.1 ± 7.9 Gy and 32.8 ± 8.8 Gy in IMRT-N and IMRT-S, respectively (*p* < 0.001). V15, V30, and V45 of tongue were significantly lower in IMRT-S (85.3 ± 15.0%, 50.6 ± 16.2%, 24.3 ± 16.0%, respectively) than IMRT-N (94.4 ± 10.6%, 64.7 ± 16.2%, 34.0 ± 18.6%, respectively) (all *p* < 0.001). Positional offsets of tongue during the course of IMRT-S was –0.1 ± 0.2 cm, 0.01 ± 0.1 cm, and –0.1 ± 0.2 cm (vertical, longitudinal, and lateral, respectively).

**Materials and Methods:**

13 patients with HNSCC underwent CT-simulations both with a neutral tongue position and a stick-out tongue for IMRT planning (IMRT-N and IMRT-S, respectively). Planning objectives were to deliver 70 Gy, 63 Gy, and 56 Gy in 35 fractions to 95% of PTVs. Radiation Therapy Oncology Group (RTOG) recommended dose constraints were applied. Data are presented as mean ± standard deviation and compared using the student *t*-test.

**Conclusions:**

IMRT-S for patients with HNSCC who refused or could not tolerate an IOD has significant decreased radiation dose to the tongue than IMRT-N, which may potentially reduce RT related toxicity in tongue in selected patients.

## INTRODUCTION

Radiation therapy (RT) is part of the standard treatment for head and neck squamous cell carcinoma (HNSCC) together with surgery and chemotherapy [1]. Despite excellent rates of locoregional cancer control, organ preservation rates, and improved survival with RT [2–4], treatments with RT or CCRT for patients with HNSCC frequently cause treatment related toxicities which include pain and dry mouth, taste changes, and difficulty swallowing during and after treatments [5, 6]. These treatments related toxicities in patients with HNSCC can adversely affect daily quality of life and nutritional status. In addition, these adverse effects often lead to inadequate nutrition, unexpected treatment breaks, and prolonged overall treatment time resulting in poor prognosis in cancer patients [7–10].

Radiation dose to salivary glands and mucosa lining in oral cavity and pharynx is closely related with decreased salivary flow, oral mucositis and dysphagia, which are exacerbated by concurrent chemotherapy during RT [10, 11]. Advances in RT techniques, such as salivary sparing Intensity-modulated RT (IMRT), has significantly preserved in salivary glands function in patient with HNSCC after RT [12, 13]. Pharyngeal constrictors (PC) sparing IMRT also improved swallowing function after RT or CCRT for HNSCC [14, 15].

The oral cavity (OC) and its subsites, such as tongue, palate and buccal mucosa, contain minor salivary glands, taste receptors, and muscles coordinating speech and swallowing [13, 16, 17]. Besides radiation dose to the entire OC, radiation exposure to tongue itself can adversely effect on saliva production, sensation of tastes, speech, and swallowing [13, 15, 18–20]. To minimize radiation dose to tongue, intraoral devices (IOD) such as bite-block, tongue blade, or customized mouthpiece has been applied to displace and away tongue from RT targets during RT or CCRT for the patients with HNSCC [21, 22]. However, applying IOD to patients with HNSCC during RT can be limited by certain medical conditions such as trismus, severe gag reflex, or discomfort from holding an IOD.

The tongue is a mobile organ and can be easily elongated without causing discomfort even in patients with trismus or gag reflex. In the present cases report, authors demonstrate anatomical changes in OC and its subsites with different tongue positions, and dosimetric advantages by applying IMRT-S over IMRT-N in patients with HNSCC but refused or did not tolerate an IOD during RT.

## RESULTS

### Patient characteristics

**Figure 1:**
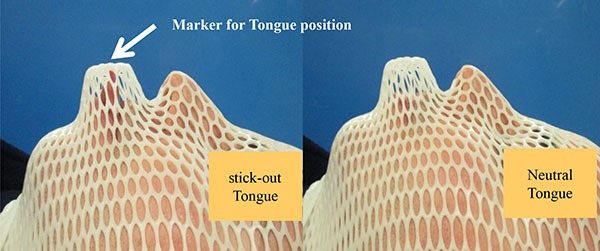
Customized thermoplastic mask for CT-simulation

Of 13 patients, 8 patients were diagnosed with squamous cell carcinoma in oropharynx without tongue involvement. Trismus, severe gag reflex, and refusal to use IOD were the reasons why patients refused or did not tolerate IOD during CT-simulation with customized immobilization mask (Figure 1). After initial observations of IMRT-S plan from patients with oropharyngeal cancer, authors in this cases report included five patients with laryngeal cancer without tongue involvement for dosimetric comparison. All patients were stage III-IVB according to the American Joint Committee on Cancer 7th staging. Radiation therapy (RT) concurrently with chemotherapy (weekly cetuximab or cisplatin every 3 week) was given to all patients. Two patients with laryngeal cancer received postoperative adjuvant RT concurrently with weekly cetuximab. Patient characteristics are shown in Table 1.

**Table 1:** Patient characteristics

Case	Primary site	Stage	Aim of RT	Reason for not use IOD
1	Tonsil	T4aN2cM0	Definitive	Trismus
2	Tonsil	T2N2bM0	Definitive	Refuse
3	Tonsil	T4bN2bM0	Definitive	Gag reflex
4	Tonsil	T4bN1M0	Definitive	Gag reflex
5	Tonsil	T4bN2cM0	Definitive	Refuse
6	Tonsil	T4aN2cM0	Definitive	Refuse
7	Tonsil	T3N3M0	Definitive	Refuse
8	Tonsil	T2N2bM0	Definitive	Gag reflex
9	Glottis	pT3N2cM0	Adjuvant	-
10	Epiglottis	T2N1M0	Definitive	-
11	Epiglottis	T2N1M0	Definitive	-
12	Epiglottis	T2N0M0	Definitive	-
13	Epiglottis	pT4aN2aM0	Adjuvant	-

Stage: American Joint Committee on Cancer 7th staging; RT: radiation therapy; IOD: intraoral devices such as bite block, tongue blade, or customized mouthpiece.

### Geometrical changes in oral cavity shape with different tongue positions

**Figure 2:**
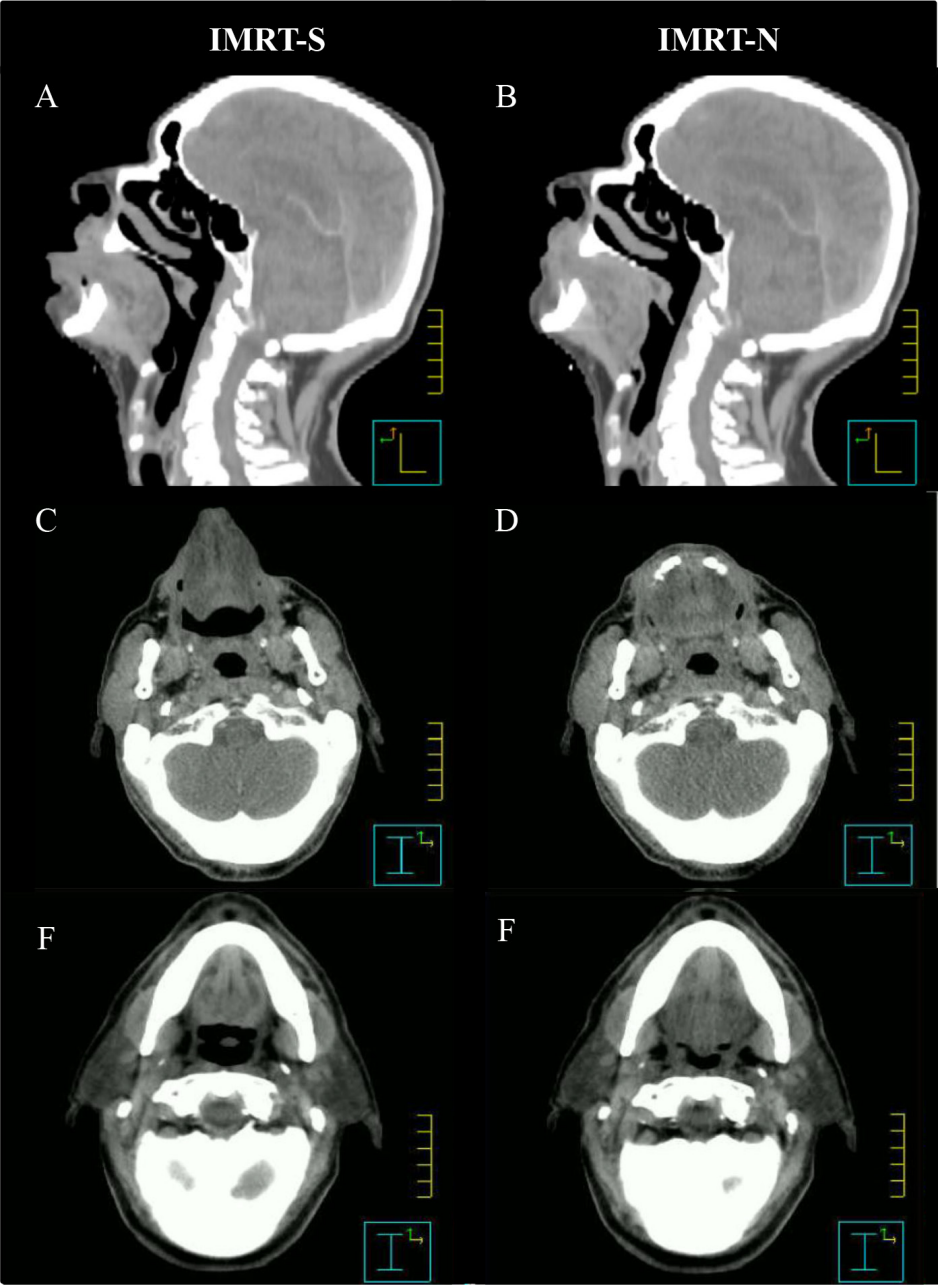
Changes in oral cavity and its subsites with different tongue position *Abbreviations:* IMRT-N = Intensity Modulated Radiation Therapy with Neutral tongue position; IMRT-S = Intensity Modulated Radiation Therapy with “Stuck-Out” tongue position; A, C, E = Images form CT-simulation with “stick-out” tongue; B, D, F = Images form CT-simulation with neutral tongue position.

The mean OC volumes were similar in IMRT-N and IMRT-S (129.2 ± 34.0 cm^3^ and 130.8 ± 38.8 cm^3,^ respectively, *p* = 0.25). However, geometrical shapes in OC changed with different tongue positions. As shown in Figures 1 and 2, patient's tongue was elongated to be out of mouth in IMRT-S. The length of tongue outside of the mouth from anterior surface of the lip was 2.2 ± 0.5 cm with “stick-out” tongue position. Entire tongue was inside of the mouth with neutral tongue position. Mean tongue volume in outside of mouth was 7.1 ± 3.8 cm^3^ with “stick-out” tongue position, which comprised 5.4 ± 2.6% of entire OC volume and 7.8 ± 3.1% of oral tongue volume. Positional offsets of tongue during the course of IMRT-S were –0.1 ± 0.2 cm in vertical, 0.01 ± 0.1 cm in longitudinal, and –0.1 ± 0.2 cm in lateral direction.

By “stick-out” tongue, lip was pushed away from gingiva in mandible. Distances between anterior surface of mandible and anterior tip of lip was 0.9 ± 0.4 cm in IMRT-N and 1.5 ± 0.3 cm in IMRT-S (*p* < 0.001). Lip was located 0.7 ± 0.3 cm more anteriorly in IMRT-S than IMRT-N.

Different tongue positions during CT-simulation also created different shapes in inside of OC. The dorsal surface of tongue was abutting the hard palate with 0.1 ± 0.2 cm of separation in IMRT-N (Figure 2B). In IMRT-S, however, there was 0.9 ± 0.3 cm of distance between the dorsal surface of tongue and hard palate (Figure 2A) (*p* < 0.001).

Interestingly, BOT was also moved to anterior direction with “stick-out” tongue resulting more distance between BOT and pharyngeal constrictor (PC) in IMRT-S (Figure 2A and 2F) than in IMRT-N (Figure 2B and 2E). The distance from posterior edge of BOT to anterior surface of PC at the level of middle of the second cervical vertebra was 2.0 ± 0.6 cm and 1.5 ± 0.5 cm with IMRT-S and IMRT-N, respectively (*p* < 0.001). Table 2 and Figures 1 and 2 demonstrate geometrical changes in OC and oropharynx with different tongue positions.

**Table 2:** Geometrical changes in oral cavity shape with different tongue positions

	IMRT-N	IMRT-S	*p*-value
Oral cavity volume	129.2 ± 34.0 cm^3^	130.8 ± 38.8 cm3	0.25
Base of tongue to pharyngeal constrictor	1.5 ± 0.5 cm	2.0 ± 0.6 cm	< 0.001
Dorsal surface of oral Tongue to palate	0.1 ± 0.2 cm	0.9 ± 0.3 cm	< 0.001
Tongue volume outside of oral cavity	0 cm^3^	7.1 ± 3.8 cm3	-
Gingiva to anterior tip of lips	0.9 ± 0.4 cm	1.5 ± 0.3 cm	< 0.001

IMRT-N: Intensity Modulated Radiation Therapy with neutral tongue position; IMRT-S: Intensity Modulated Radiation therapy with “stick-out” tongue position; *p*-value using *t*-test, Data are presented as mean ± standard deviation.

### Radiation dose to oral cavity and its subsites with different tongue positions

Changes in geometrical shapes in OC with different tongue position during CT-simulations have affected on radiation dose to OC and its subsites in IMRT plans. Although mean OC volumes were similar in IMRT-N and in IMRT-S (Table 2), there was 11.1 ± 6.9% of reduction in D_mean_ to OC with IMRT-S (31.4 ± 8.7 Gy) comparing to IMRT-N (34.9 ± 8.0 Gy) (*p* < 0.001). OC volume receiving equal or greater than 36 Gy (V36) was 40.6 ± 16.9% and 33.0 ± 17.0% of entire OC (IMRT-N and IMRT-S, respectively, *p* < 0.001).

**Figure 3:**
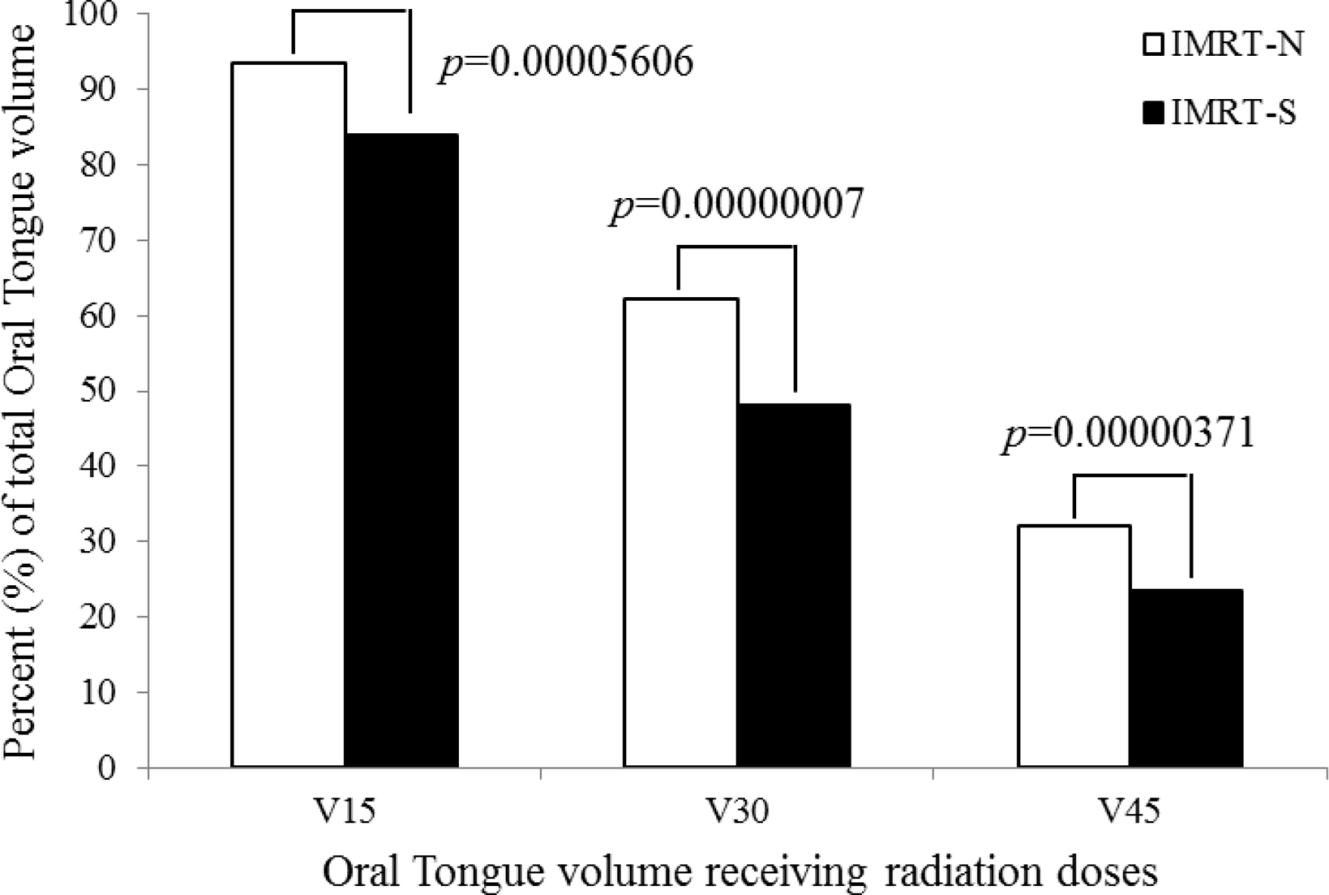
Oral tongue volume receiving radiation *Abbreviations*: IMRT-N = Intensity Modulated Radiation Therapy with Neutral tongue position; IMRT-S = Intensity Modulated Radiation Therapy with “Stuck-Out” tongue position; V15, V30, and V45 = volume receiving ≥ 15 Gray (Gy), 30 Gy, and 45 Gy; *p*-value using *t*-test.

By elongated and stretched out tongue from mouth, there were significant decreases in radiation dose to tongue in IMRT-S compared to those in IMRT-N. D_mean_ to tongue was decreased from 38.1 ± 7.9 Gy in IMRT-N to 32.8 ± 8.8 Gy in IMRT-S (14.8 ± 7.3% of reduction) (*p* < 0.001). Figure 3 shows the volume of tongue receiving a dose ≥ 15, 30, and 45 Gy (V15, V30, and V45) were significantly lower in IMRT-S (85.3 ± 15.0%, 50.6 ± 16.2%, 24.3 ± 16.0%, respectively) than IMRT-N (94.4 ± 10.6%, 64.7 ± 16.2%, 34.0 ± 18.6%, respectively) (all *p* < 0.001).

Changes in lips positions with different tongue positions also demonstrated that 16.0 ± 10.8% of reduction in D_mean_ to lips with IMRT-S comparing to IMRT-N. D_mean_ to lips was 16.0 ± 3.7 Gy in IMRT-N and 13.5 ± 4.0 Gy in IMRT-S (*p* < 0.001).

Decrements in radiation dose to OC and its subsites with IMRT-S over IMRT-N were observed both in oropharyngeal (*n* = 8) and laryngeal cancer (*n* = 5) patients (Table 3).

**Table 3:** Mean Radiation dose (D_mean_) to oral cavity and its subunits

Primary		Oral cavity	*p*-value	Oral Tongue	*p*-value	Lips	*p*-value
Tonsil (*n* = 8)	IMRT-N	39.3 ± 5.6 Gy		42.5 ± 5.3 Gy		15.8 ± 4.2 Gy	
	IMRT-S	35.9 ± 6.2 Gy	< 0.001	37.6 ± 6.2 Gy	< 0.001	13.2 ± 4.5 Gy	< 0.001
Larynx (*n* = 5)	IMRT-N	27.9 ± 6.2 Gy		30.9 ± 5.4 Gy		16.8 ± 0.3 Gy	
	IMRT-S	24.2 ± 7.3 Gy	< 0.001	25.2 ± 6.2 Gy	< 0.001	14.5 ± 1.6 Gy	0.1282
Total (*n* = 13)	IMRT-N	34.9 ± 8.0 Gy		38.1 ± 7.9 Gy		16.0 ± 3.7 Gy	
	IMRT-S	31.4 ± 8.7 Gy	< 0.001	32.8 ± 8.8 Gy	< 0.001	13.5 ± 4.0 Gy	< 0.001

IMRT-N: Intensity Modulated Radiation Therapy with Neutral tongue position; IMRT-S: Intensity Modulated Radiation Therapy with “stick-out” tongue position; Primary: Primary site of caner; Gy: gray; *p*-value using *t*-test, Data are presented as mean ± standard deviation.

## DISCUSSION

Oral cavity (OC) contains most of the minor salivary glands, which are mostly located in the buccal, labial, distal palatal, and lingual mucosa [24]. The minor salivary glands has groups of secretory endpieces made up of mucous acinar cells and serous or seromucous demilune cells. The ductal systems are consisted with intercalated ducts, intralobular ducts, and excretory ducts opening directly through the mucosa in OC. Unlikely major salivary glands producing saliva fully only with stimulation, minor salivary glands secrete saliva into OC continuously all day and night, which is important for tissue lubrication and moisturizing inside of OC. Minor salivary glands also secrete several antimicrobial proteins and immunoglobulins into OC. Radiation dose to minor salivary glands in OC was significant predictor for dry mouth even after sparing major salivary glands with IMRT [13].

Among minor salivary glands, the lingual serous (von Ebner's) glands on the dorsal surface of the tongue secrete digestive enzymes and proteins with facilitate the perception of taste [16, 17]. Radiation dose relationship with impairment tastes during RT or CCRT in patient with HNSCC has been reported [18, 24]. Radiation-induced tastes alteration in patients with HNSCC increased rapidly after as low as radiation dose at 10 Gy and reached maximum at 40 Gy. The mechanism of tastes alteration with RT is likely related to direct damage to receptors within the tongue as the radiation doses received by these regions beyond 20~30 Gy to oral cavity [18]. As salivary gland-sparing RT techniques improve salivary functional outcomes for many patients with HNSCC, tastes impairment may become a more recognized problem for long-term survivors after RT.

Authors in this report observed 5.4 ± 2.6% of OC volume and 7.8 ± 3.1% of oral tongue volume became in outside of mouth, i.e., away from RT targets, with “stick-out” tongue position, which has significantly decreased D_mean_ to oral tongue comparing to with neutral tongue position (Table 3). Of entire OT volume, V30 was also significantly reduced from 64.7 ± 16.2% in IMRT-N to 50.6 ± 16.2% in IMRT-S (*p* < 0.001). For the patients with HNSCC and who refuses or can't use bite-block or tongue blade, IMRT-S rather than IMRT-N can lower D_mean_ to OC and OT, and lower V30 to oral tongue, and therefore, may spare more minor salivary glands as well as taste receptors on the tongue.

Changes in speech and swallowing function in patients with HNSCC after RT or CCRT are also related to radiation dose to OC, tongue and pharyngeal constrictor [11, 19, 25, 26]. Muscle weakness and fibrosis are known etiologies for post RT dysphagia. In animal study, radiation damages on the sarcoplasmic reticulum (SR) can cause lower than normal intracellular Ca^2+^, which creates less force in muscles on stimulation [27]. Radiation also decreases membrane excitability so that less muscle fiber is activated with a given stimulation [28]. Clinically, high D_mean_ to tongue was related to decrease in tongue movement, which adversely effecting on speech quality after RT [19]. Data suggest decreased oral and pharyngeal motility after RT for patients with HNSCC [11, 19, 25, 26]. The probability of dysphagia has shown to be increased 19% with every additional 10 Gy to pharyngeal constrictor muscle [25]. These reports support that lowering radiation dose to tongue and pharynx is important to minimize not only mucositis, dry mouth, alteration of taste, but also speech alteration and dysphagia during and after RT or CCRT.

In addition to decreased radiation dose to tongue with IMRT-S, this report also found an increased distance from BOT to pharyngeal constrictor from 0.9 ± 0.3 cm with neural tongue position to 1.5 ± 0.5 cm with “stick-out” tongue position (Figures 1 and 2). For the patient whose cancer involves BOT, these increased distance between BOT (i.e. radiation target) to pharyngeal constrictor (i.e. OAR) with “stick-out” tongue could potentially be of benefit for reducing radiation dose to pharyngeal constrictor. Hypothetically, decreased radiation dose to tongue and pharyngeal constrictor by “stick-out” tongue (with or without bite-block) can improve dry mouth, taste changes, speech, and swallowing function in selected patient with cancer in BOT.

Additionally, during “stick-out” tongue, lips were located at 0.7 ± 0.3 cm more anteriorly than during neutral tongue position resulting significant decrease in D_mean_ to lips by 16.0 ± 10.8% with IMRT-S than IMRT-N (*p* < 0.001). Distance from dorsal surface of oral tongue to hard palate was increased to 0.9 ± 0.4 cm with “stick-out” tongue position from 0.1 ± 0.2 cm with neutral tongue position (Figure 2). For the patient with cancer in palate but who refuses or does not tolerate an IOD during RT, those increased distance between surface of tongue and palate can reduce radiation dose to tongue.

Although confirming clinical benefits to OARs (tongue, pharyngeal constrictor, and lips) from “stick-out” tongue during RT needs further investigation, this report has showed lower radiation doses to OARs as low as reasonably achievable for the patients who refused or could not use bite-block or tongue blade during RT, comparing to neutral tongue position during RT (Table 3). With the marker for tip of tongue in customized thermoplastic mask, daily positional offsets for tongue were within acceptable ranges (vertically –0.1 ± 0.2 cm, longitudinally 0.01 ± 0.1 cm, and laterally –0.1 ± 0.2 cm) throughout the course of RT. In fact, tongue was not involved by cancer in all patients in this report. Therefore, as long as patients “stick-out” their tongue, tongue will have always more distance from radiation target without compromising radiation delivery to PTV. More distance from radiation target to tongue will decrease radiation dose to tongue.

## MATERIALS AND METHODS

Between January of 2013 and November of 2015, thirteen patients with histologically proven squamous cell carcinoma of the head and neck underwent CT-simulations: one with neutral tongue and one applying “stick-out” tongue position for planning IMRT. Due to the uncertainty of reproducible tongue position during daily RT, authors had limited applying “stick-out” tongue position only to the patients with oropharyngeal cancer but without oral tongue involvement. Therefore, daily variation of tongue position would not compromise radiation targets coverage.

Before the CT-simulation, patients were informed and guided to “stick-out” tongue with comfortable and reproducible ways. The thermoplastic mask was customized to create a marker that indicates the location of tip of the tongue in the “stick-out” tongue position (Figure 1), and immobilization the head and neck. The patients were advised to “stick-out” tongue and touch the marker in thermoplastic mask. Then patients underwent CT-simulations using customized thermoplastic mask with “stick-out” tongue and neutral tongue position separately. CT images with a 3.0 mm slice thickness and intravenous contrast was obtained from vertex to aortic arch. According to the RTOG guidelines, the following OARs were contoured for the pretreatment planning: spinal cord, brain stem, mandible, parotid gland, submandibular gland, pharyngeal constrictor (PC), larynx, upper esophagus, and brachial plexus. The delineated structure OC covered gingiva, hard palate, buccal mucosa, floor of the mouth, oral vestibule, lips, and the oral tongue (OT) including the intrinsic tongue muscles, the anterior and medial genioglossus. The base of tongue (BOT) was covered posterior genioglossus, the geniohyoid, and adjacent to suprahyoidal muscles. Gross tumor volume (GTV) was defined as all gross disease on the CT, or positron emission tomography (PET). In the radical setting, GTV was manually expanded to clinical target volume (CTV1) at the discretion of the radiation oncologist. The CTV1 was manually expanded to CTV2 to cover the high-risk regions around the primary tumor and nodal disease. The CTV3 covered low-risk lymph nodal stations. Planning target volumes (PTV1, PTV2, and PTV3) were generated with an isotropic expansion of 3 mm from CTV1, CTV2, and CTV3 respectively. A total dose of 70 Gray (Gy) to PTV1, 63 Gy to PTV2, and 56 Gy to PTV3 were prescribed to PTVs in 35 fractions, using simultaneously integrated boost technique. Planning objectives required PTVs coverage of 95% to 107%. Concerning OARs, there were set as follows: spinal cord = maximum radiation dose (D_max_) in 0.1 cc < 50 Gy; brain stem = D_max_ in 0.1 cc < 54 Gy; mandible = D_ma_x in 0.1 cc < 70 Gy, and V50 < 30%; parotid glands = mean radiation dose (D_mean_) < 26 Gy; submandibular gland = D_mean_ < 36 Gy; PC = D_mean_ < 50 Gy; larynx = D_mean_ < 36 Gy; upper esophagus = D_mean_ < 40 Gy; brachial plexus = D_max_ in 0.1 cc < 64 Gy; and OC = D_mean_ < 36 Gy. Treatment planning aimed to reduce doses to OARs as much as possible without compromising the coverage of the PTVs. Pinnacle radiation therapy planning system (version 9.4, Philips healthcare, Fitchburg, WI) was used for IMRT planning. All plans were performed with 7- or 9-beam using 6 MV photon applied using a Varian iX Silhouette (Varian Medical System, Palo Alto, CA).

Daily cone beam CT (CBCT) was performed before IMRT for image-guidance and checking tongue position. The tongue contoured on CT-simulation was compared with one on daily CBCT to measure daily offset of the tongue during the course of IMRT-S. Dosimetric comparison between IMRT-N and IMRT-S was performed using the overall target dose-volume histogram (DVH), quantitative values of the target minima, maxima, and mean dose and normal tissue mean dose.

Statistical analysis of dosimetric comparison between IMRT-N and IMRT-S was done using a Student *t*-test. Data are presented as mean ± standard deviation. A probability level of a *p* value of < 0.05 was considered significant.

## CONCLUSIONS

In this cases report, IMRT-S for patients with HNSCC but who refused or could not tolerate an IOD have significant decreased radiation dose to OC and its subsites, specifically oral tongue and lips, and increased distance between base of tongue and pharyngeal constrictor, dorsal surface of tongue to palate comparing to IMRT-N. With customized thermoplastic mask, daily “stick-out” tongue position was reproducible. These dosimetric benefits with IMRT-S are noticed in both oropharyngeal and laryngeal primary HNSCC. To confirm decreased radiation doses to OARs with IMRT-S translating into clinical benefits, prospective trial with long-term clinical outcome is warranted.
